# Identification and validation of SNP markers linked to seed toxicity in *Jatropha curcas* L

**DOI:** 10.1038/s41598-019-46698-4

**Published:** 2019-07-15

**Authors:** Daniele Trebbi, Samathmika Ravi, Chiara Broccanello, Claudia Chiodi, George Francis, John Oliver, Sujatha Mulpuri, Subhashini Srinivasan, Piergiorgio Stevanato

**Affiliations:** 1 Syngenta Seeds Inc., California, USA; 2 DAFNAE, Università degli Studi di Padova, Legnaro, Italy; 3 JATROPOWER AG, Baar, Switzerland; 4 ICAR, Indian Institute of Oilseeds Research, Hyderabad, India; 5 IBAB, Institute of Bioinformatics and Applied Biotechnology, Bangalore, India

**Keywords:** Sequencing, DNA replication

## Abstract

Edible/non-toxic varieties of *Jatropha curcas* L. are gaining increasing attention, providing both oil as biofuel feedstock or even as edible oil and the seed kernel meal as animal feed ingredient. They are a viable alternative to the limitation posed by the presence of phorbol esters in toxic varieties. Accurate genotyping of toxic/non-toxic accessions is critical to breeding management. The aim of this study was to identify SNP markers linked to seed toxicity in *J. curcas*. For SNP discovery, NGS technology was used to sequence the whole genomes of a toxic and non-toxic parent along with a bulk of 51 toxic and 30 non-toxic F_2_ plants. To ascertain the association between SNP markers and seed toxicity trait, candidate SNPs were genotyped on 672 individuals segregating for seed toxicity and two collections of *J. curcas* composed of 96 individuals each. *In silico* SNP discovery approaches led to the identification of 64 candidate SNPs discriminating non-toxic and toxic samples. These SNPs were mapped on Chromosome 8 within the Linkage Group 8 previously identified as a genomic region important for phorbol ester biosynthesis. The association study identified two new SNPs, SNP_J22 and SNP_J24 significantly linked to low toxicity with R^2^ values of 0.75 and 0.54, respectively. Our study released two valuable SNP markers for high-throughput, marker-assisted breeding of seed toxicity in *J. curcas*.

## Introduction

*Jatropha curcas* L. is being fostered as a sustainable source of bioenergy and food. The most valuable parts of the plant are the kernels containing high amounts of oil and protein suitable for creating a range of beneficial products^1^. However, the phorbol esters in the toxic varieties of *Jatropha* render the most important byproduct of the biofuel extraction, the seed kernel meal, unsuitable for consumption. The adverse effects of toxicity have been firmly established on microorganisms to higher animals using extracts from fruit, seed, oil, roots, latex, bark, and leaves^2^. This affects the benefits of the plant negatively.

The identification of non-toxic edible *Jatropha* varieties presents a more suitable source of oil and animal feed ingredients^3^. An interesting study compared the two varieties for growth pattern, pest incidence and seed productivity^4^. It has been confirmed that variation in edibility of *Jatropha* seeds is due to a single trait i.e. the presence of phorbol esters. The content of other anti-nutrients such as curcin in the seeds of toxic and edible *J. curcas* remain unchanged^3,5^. Another study showed negligible waste production from Mexican non-toxic variety with use of both biodiesel and de-oiled seed cake as a protein and carbohydrate source^6^. Commercial breeders also value the generation of non-toxic accessions bypassing the chemical detoxification of the toxic varieties. Nevertheless, the seed characteristic is the single most determinant of the target market. This necessitates molecular markers strongly distinguishing the two varieties to aid in breeding. Efforts in this direction include generation of SCAR markers specific for toxic and non-toxic plants^7^. Studies have identified seven polymorphic microsatellite markers using RAPD and AFLP techniques^8^. Another group identified microsatellite markers classifying non-toxic and toxic *J. curcas* correlating them to the phorbol ester (PE) levels^9^. The generation of the first genetic map using SNP markers and also the identification of a locus for phorbol ester biosynthesis has been extremely valuable^10^. In addition, a high-resolution linkage map has recently been made available aiding in the mapping of important agronomic traits^11^.

More recently, SNP based markers are dominating the molecular breeding field due to i) their abundance in plant genomes ii) high-throughput detection in NGS and genotyping platforms iii) lower false positives due to bi-allelic nature iv) availability of a range of computational pipelines for SNP calling v) clear read-off in haplotypes from inbred lines^12–14^. Their wide spread application in the breeding of model plants has been well documented^15–18^. They continue to be the marker of choice for candidate gene identification, trait discrimination and diversity analysis. In the past decade, with the availability of affordable and high-throughput technologies such as NGS, the same approach has been applied to whole genomes of non-model plants^19–21^.

The objective of the present study was to identify SNP markers linked to seed toxicity in *J. curcas*. A thorough experimental design for sequencing (toxic JPT-86 parent, non-toxic JPNT-2 parent and F_2_ *Jatropha* plants from crossing JPNT-2 X JPT-86) enabled the identification of putative SNP markers governing seed toxicity. The results reported in this study demonstrate the first use of OpenArray technology for rapid screening of candidate SNPs in *J. curcas* along with PyroMark assays for accurate SNP identification. The reliability of SNPs from the associations study have been verified on two independent datasets. These SNP markers, SNP_J22 and SNP_J24 as well as the genotyping assay will be beneficial to the breeding of *J. curcas* non-toxic varieties.

## Results

### Phenotyping

**Figure 1:**
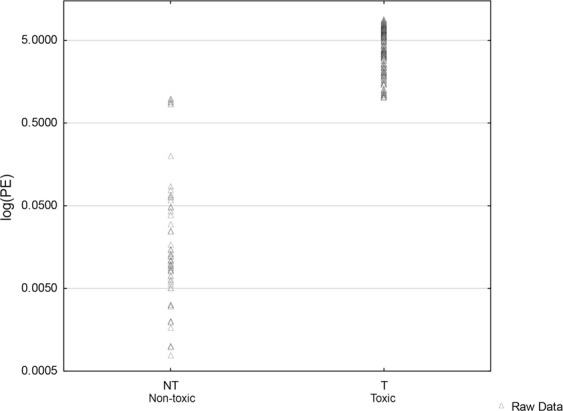
Scatter plot of log(PE)/phorbol ester (PE) content vs toxicity status in F_2_ population segregating for seed toxicity and generated by crossing a high yielding, early maturing, non-toxic edible accession from Mexico (JPNT-2) with a toxic, non-edible accession from India (JPT-86).

Figure 1 shows the phorbol ester content (averaged over duplicates) in 249 plants from the F_2_ population segregating for seed toxicity and used for association study. The estimation of phorbol ester content by HPLC allowed for categorization of the plants as toxic and non-toxic. A level of 0.1 mg/g has been routinely considered to be the upper limit of phorbol ester levels beyond which test animals start rejecting diets containing toxic jatropha seeds^4^. The method is also not sensitive below the mentioned level of phorbol esters.

### SNP discovery and mapping

**Figure 2:**
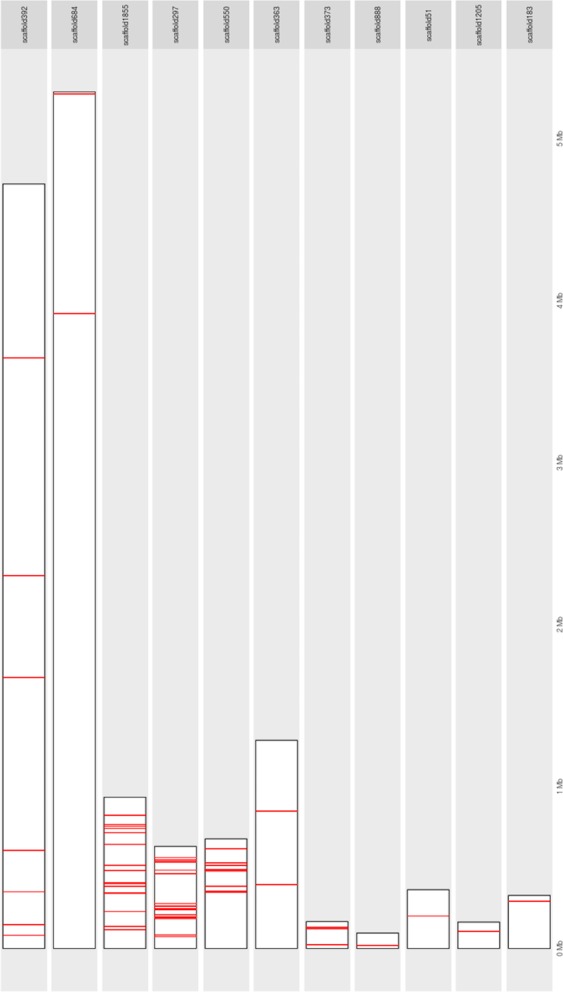
Physical positions of 64 toxicity related SNPs on Wu *et al*. scaffolds (2015).

SNP analysis from whole genome sequencing of parental DNA revealed a total of 6,248 homozygous SNPs between the two parental lines of *J. curcas*. Comparative analysis of the SNP data between toxic and non-toxic segregating bulks for the 6,248 homozygous positions yielded a total of 64 SNPs. These SNPs that clearly differentiated the two bulks were further considered for experimental validation. The SNP markers mapped on 41 Kazusa scaffolds identifying putative genomic candidate sequences linked to the gene(s) influencing toxicity in *J. curcas* (Supplementary Material S2). The 64 candidate SNPs mapped onto 11 scaffolds of the genome provided by Wu *et al*. (2015), specifically between 10 to 15 Mbp on the upper arm of chromosome 8 (Fig. 2). In this study, Kazusa genome (JAT_r4.5) was used as reference genome and the SNP analysis revealed that the toxic parental line sequenced here contains 6,057 SNPs (97%) matching the reference (Kazusa) allele and only 191 SNPs (3%) had the alternative allele.

### Association study

**Figure 3:**
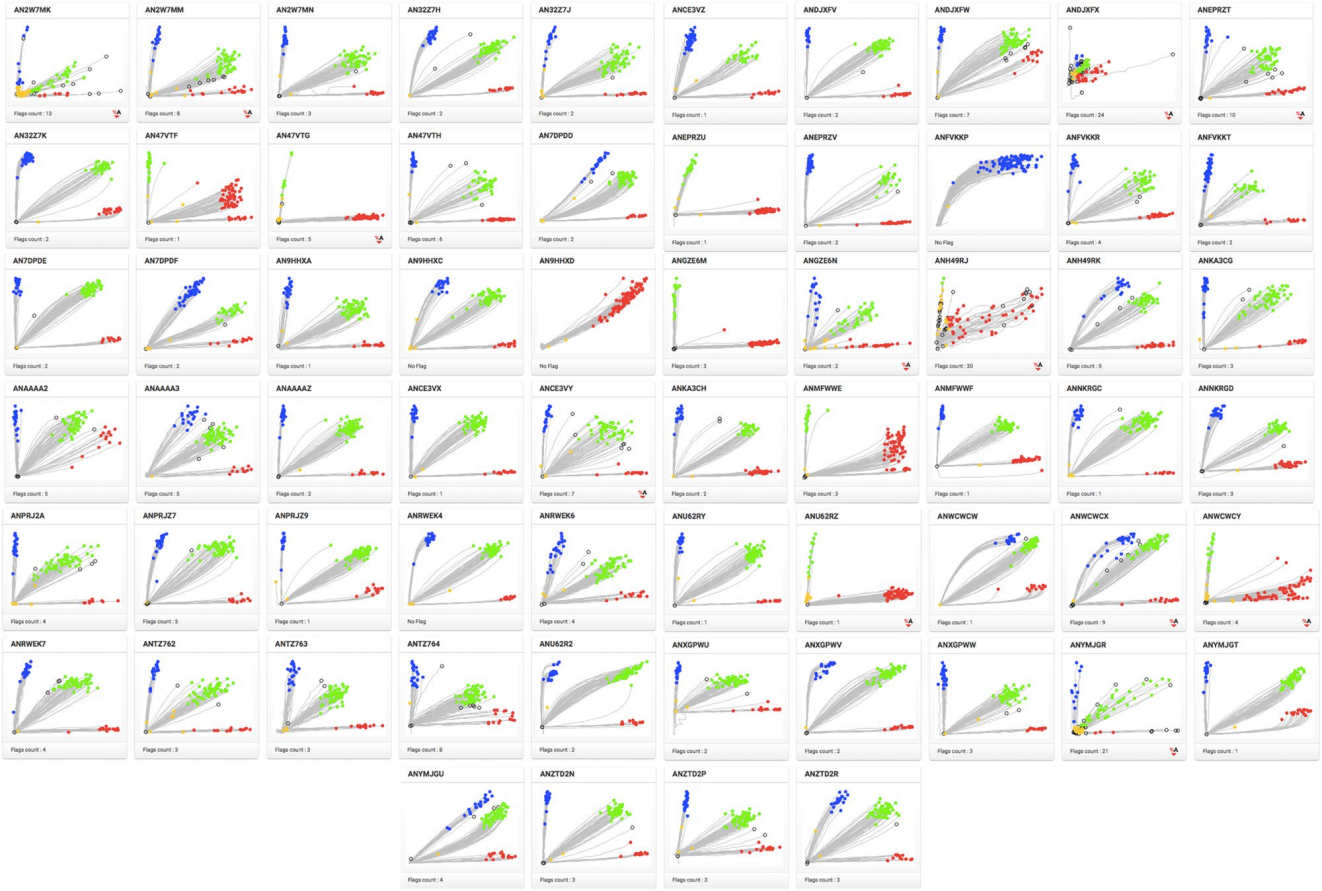
Allelic discrimination plots of 64 candidate SNPs genotyped on *J. curcas* samples using OpenArray technology.

**Figure 4:**
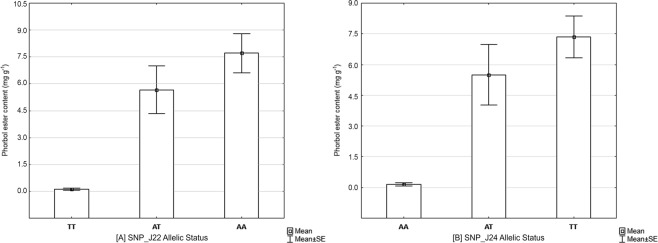
Effect of SNP_J22 and SNP_J24 allelic status on phorbol ester content. The homozygous T/T genotype for SNP_J22 and A/A genotype for SNP_J24 is determining the lowest phorbol ester contents in evaluated samples.

The collection of 672 plants comprising toxic and non-toxic plants were genotyped with a panel of 64 SNPs using OpenArray technology. TaqMan primers and probes sequences are available in Supplementary Material S3. Allelic discrimination plots obtained from screening the 64 candidate SNPs on OpenArray platform is shown in Fig. 3. The three distinct clusters are representative of the samples presenting three different genotypes. The OpenArray genotyping plates showed a genotyping call average success rate of 93%. The association between three SNP genotypes and phorbol ester content was tested by fitting one SNP at a time in a linear regression model. Regression analysis allowed the identification of two SNPs, SNP_J22 and SNP_J24 significantly linked to low toxicity with R^2^ values of 0.75 and 0.54, respectively (Fig. 4). The physical position of these highly significant SNPs, SNP_J22 and SNP_J24 are on the same scaffold Jcr4S00944 of the Kazusa genome (JAT_r4.5) at positions 3,558 bp and 25,943 bp respectively. The same SNPs were mapped to scaffold297 of Wu *et al*.’s genome at precise locations 184,697 bp for SNP_J22 and 207,326 bp for SNP_J24.

**Figure 5:**
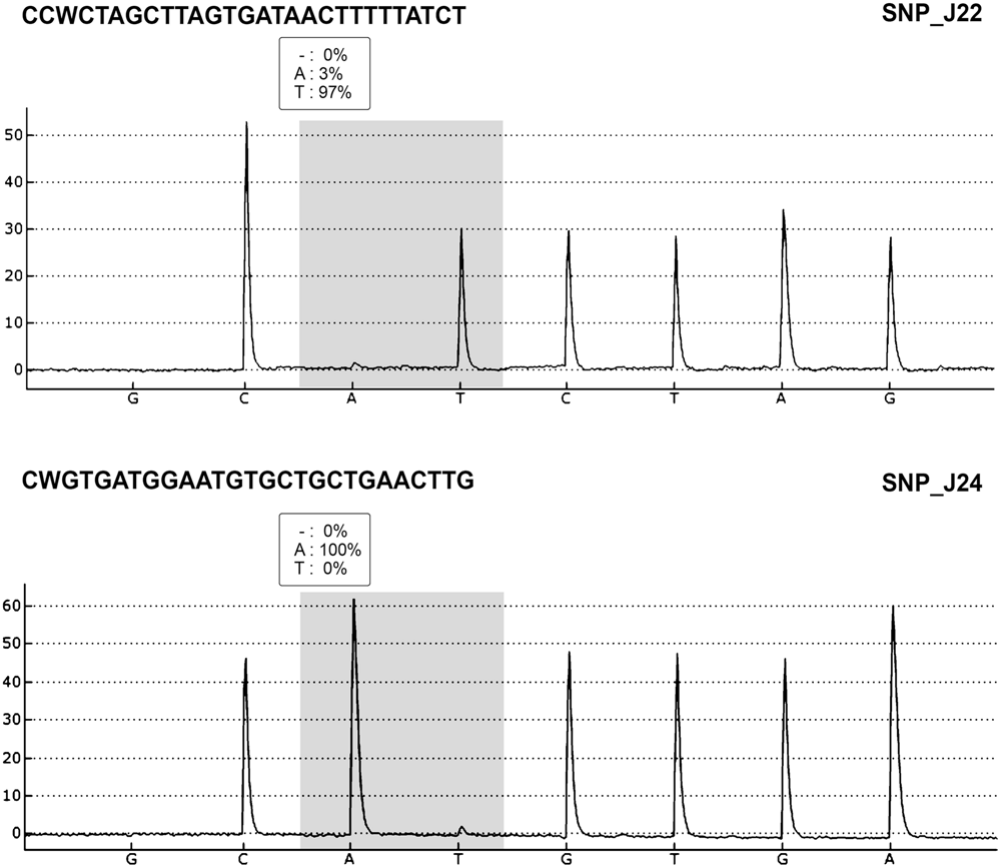
Pyrograms showing polymorphisms in the SNP_J22 and SNP_J24 markers obtained with PyroMark Allelic Quantification Assays.

In addition, to F_2_ population, a set of toxic and non-toxic accessions (Supplementary Material S1) was used to further ascertain the association between toxicity trait and markers identified in this study. Pyrosequencing Allele Quantification assays were designed to detect SNP_J22 and SNP_J24 on large segregating populations for seed toxicity. Sequences of primers for pyrosequencing assays are available in Supplementary Material S4. Representative pyrograms indicating the polymorphism in the SNP_J22 and SNP_J24 markers are presented in Fig. 5. Signals from the analyzed DNA sequence are represented as peaks in the pyrogram, corresponding to the number of identical nucleotides incorporated (Fig. 5). The association between the two SNPs and low toxicity character was also evaluated and confirmed using chi-square statistic (Table 1).

**Table 1:** Allele frequencies of SNP_J22 and SNP_J24 from pyrosequencing allelic quantification tested in two validation datasets.

SNP_J22			T	A	*X* ^2^	*p* value
Test dataset 1	Parent	Non-toxic	100%	0		
		Toxic	0	100%		
	F_2_	Non-toxic (n = 56)	44 (78.5%)	12 (21.4%)	87.0964	<0.0001
		Toxic (n = 546)	111 (20.3%)	435 (79.6%)		
Test dataset 2	Diverse germplasm	Non-toxic (n = 48)	31 (64.6%)	17 (35.4%)	22.8448	<0.0001
		Toxic (n = 48)	8 (16.7%)	40 (83.3%)		
**SNP_J24**			**A**	**T**	***X*** ^**2**^	***p*** **value**
Test dataset 1	Parent	Non-toxic	100%	0%		
		Toxic	0%	100%		
	F_2_	Non-toxic (n = 56)	42 (75%)	14 (25%)	92.6161	<0.0001
		Toxic (n = 546)	95 (17.3%)	451 (82.6%)		
Test dataset 2	Diverse germplasm	Non-toxic (n = 48)	33 (68.8%)	15 (31.3%)	31.4818	<0.0001
		Toxic (n = 48)	6 (12.5%)	42 (87.5%)		

Table reports the *X*^2^ square and the respective *p* value. Toxic and non-toxic parents were also analyzed as controls.

## Discussion

*Jatropha curcas* is an extraordinary industrial crop with compelling uses. Mismanagement of its valued byproducts is regarded unsustainable. Further, plantations of non-toxic *J. curcas* potentially increase the revenue by 25% compared to toxic varieties (Jatropower AG, Switzerland). Phorbol ester remains a crucial trait for research and in the assignment of cultivars to specific target markets. Therefore, methodologies allowing identification and improvement of non-toxic *J. curcas* is pivotal to its breeding and domestication.

The availability of existing genomic data in *Jatropha*^22,23^ along with a comprehensive experimental design and a relatively simple genome made the genome wide discovery of SNP markers related to toxicity straightforward. Comparative studies between the two genomes identified the cultivar sequenced by the Kazusa DNA Research Institute to be similar to a toxic line. This information is also beneficial to the breeders working on genetic improvement of this particular cultivar.

SNP discovery from whole genome sequencing revealed 64 candidate SNPs in Linkage group 8, a locus previously identified for phorbol ester biosynthesis and toxicity related genes^10^. The sequences of the 64 SNPs linked to toxicity are provided in Supplementary Material S2 for further analyses. Recently, an updated assembly with linkage map was made available^11^. The localization of all 64 SNPs in the upper arm of chromosome 8 suggests the identification of a genomic region that regulates toxicity. However, due to the limited recombination events expected in a F_2_ population and number of plants tested, the candidate genomic region still spans several kilo base pairs. Furthermore, correlating the SNPs with predicted genes can shed light on putative biosynthetic pathways influencing the toxicity trait.

The basis of association analysis is screening of large number of SNPs on large number of samples and thus, the use of OpenArray technology in this study is pertinent. This technology offers several advantages as demonstrated in this study: (i) low cost of € 0.07 per sample, (ii) accurate genotyping with an average call rate of 93% and (iii) rapid genotyping allowing generation of 36,864 data points in a day. SNP_J22 and SNP_J24 obtained from the association study are a fundamental step in the introduction and cultivation of non-toxic *J. curcas* accessions. This study is the first demonstration of SNP screening using OpenArray platform in *J. curcas*. The SNPs present a high correlation with the low phorbol ester content. The reproducibility of results using this tool has also been confirmed in prior studies^24,25^. The success of an association study is realized through translation of identified SNPs for routine genotyping in breeding. This process involves testing thousands of samples for an extremely critical trait like toxicity, with high confidence using as few characteristic markers. Pyrosequencing has earlier been shown as an appropriate method for SNP analysis where allele frequency is 50% and when the nucleotide variants are clearly known^26^.

Our study confirms the accuracy and reliability of allelic quantification accomplished with PyroMark. The assay design is simple and also allows the sequencing of bases around the SNP. This gives more information about the PCR product analysed and its authentication^27^. It allows allelic quantification at a cost of € 12 per assay. The economic feasibility is apt considering the widespread cultivation of *J. curcas* in countries with limited fiscal capacity. Further, the results of this study were collated from validation on samples representing diverse cultivated and wild germplasms. Recent studies have shown the wide genetic variability specially in the germplasms lacking phorbol esters among accessions from Mexico and Central America^28^. These germplasms could be segregating for multiple traits including toxicity. Therefore, an accurate SNP detection step subsume characterization of accessions and fixation of lines. In our study, Pyrosequencing technology clearly allowed separation of the true homozygotes from heterozygotes. The above reasons detailed enhance the applicability of Pyrosequencing in breeding. To our knowledge, this is the first research adopting allelic quantification by Pyrosequencing in *J. curcas*.

SNP markers along with the modern genomics approaches and NGS technologies accelerate the improvements in molecular-genomics-assisted plant breeding. As shown in the present study, these SNPs can be combined with other known SNPs^10^ related to toxicity with an aim to (i) create a SNP panel sanctioning the discrimination of non-toxic varieties in a mixed pool (ii) check contamination prior to usage as animal feed and (iii) fingerprint samples for identity and diversity analysis. A handful of SNP markers can significantly improve the accuracy and efficiency of trait selection in germplasm management^19,29^.

## Material and Methods

### Plant materials

An F_2_ population segregating for seed toxicity trait was generated by crossing a high yielding, early maturing, non-toxic edible accession originating from Mexico (JPNT-2) with a toxic, non-edible accession from India (JPT-86). In addition to the F_2_ population, a set of toxic and non-toxic accessions from various countries were used to further ascertain the association between toxicity trait and markers identified in this study. A list of accessions used in this study with their country of origin and toxicity status has been provided in Supplementary Material S1. All plant materials were raised at an experimental farm near Coimbatore, Tamil Nadu state, India (Latitude: 10.764972°; Longitude: 79.737439°) at a plant to plant and row to row spacing of 2.0 m each.

### Phenotyping

Seeds from the F_2_ population of plants were used for extraction and quantification of phorbol ester content by high performance liquid chromatography (HPLC). Each plant was analyzed in duplicate. The results are expressed as equivalent to a standard phorbol-12-myristate13-acetate in milligrams per gram (mg/g). HPLC conditions for estimation of phorbol ester content was carried out in accordance with previously described research^30,31^. An average phorbol ester cut off of 0.1 mg/g was used to categorize toxic plants from non-toxic plants^4^.

### DNA isolation

Leaves from the parents and F_2_ progeny was used to extract DNA. This was performed on the BioSprint 96 platform (Qiagen) using the BioSprint 96 DNA Plant Kit (Qiagen) following the method described in our previous study^32^. DNA of 51 toxic samples, 30 non-toxic individual samples, 1 toxic parent and 1 non-toxic parent were used downstream for sequencing.

### Library preparation for whole genome sequencing

Sequencing libraries were prepared by using TruSeq DNA HT Kit (cat# FC-121-2003, Illumina). The standard protocol recommended by the company was followed for library preparation. The quality and molarity of the libraries was evaluated by TapeStation (Agilent Technologies). Bulks were composed of 51 and 30 normalized DNA samples for the toxic and non-toxic bulks, respectively. Parental DNA samples were barcoded individually. All the samples were sequenced on the HiSeq2500 system (Illumina) using paired-read technology of 100 bp.

### SNP Identification and mapping analysis

All the sequenced reads were checked for their quality using FastQC v0.11.4^33^. The sequenced reads were then aligned to the reference genome from Kazusa DNA Research Institute (Version 4.5; http://www.kazusa.or.jp/jatropha/) using bowtie2 v2.2.1-0^34^ and the alignment files were further processed with SAMtools v1.4^35^. PCR duplicates were marked using Picard tools v2.6^36^. Variant calling was done using bcftools call v1.4^37^. SNP identification was done considering the following four groups: toxic parental; non-toxic parental; toxic bulk and non-toxic bulk. Only homozygous SNPs present with different allelic status between the parents were selected. For bulk segregant analysis, among the selected SNPs only those segregating with the parental non-toxic allele frequency >0.95 in the non-toxic bulk (expected = 1.00) and <0.50 in the toxic bulk (expected = 0.33) were selected. For the SNPs segregating between the bulks, which are the candidate SNPs linked to the gene(s) of interest, sequences harboring the SNPs were mapped against Kasuza scaffolds (Version 4.5; http://www.kazusa.or.jp/jatropha/) and the newly developed draft genome by Wu *et al*.^11^ to identify their genomic locations using BLAST^38^. The physical location of toxicity related SNPs on Wu *et al*.’s genome was visualized using a customized R script.

### Association study

For association study, a collection of 672 F_2_ plants comprising toxic and non-toxic plants were genotyped with discovered SNPs using the Quant Studio 12 K Flex Real Time PCR System and OpenArray platform (Life Technologies). Sample preparation and loading methods were performed following the method described previously^39^. Selected SNPs showing best discrimination were further genotyped using PyroMark Q48 Advance Reagents on the PyroMark Q48 (Qiagen) on a collection of 602 F_2_ plants and 96 plants of diverse germplasm. PCR and sequencing primers were developed using the PyroMark Assay Design Software 2.0.1.15 (Qiagen). The following thermal cycling conditions were used: 15 min at 95 °C, followed by 45 cycles of 30 s at 94 °C, 30 s at 60 °C, 30 s at 72 °C and 10 min at 72 °C. Sequence analysis was performed using the PyroMark Q48 Autoprep software (Version 2.4.2) in the Allele Quantification mode. The association between three SNP genotypes and toxicity character was tested by fitting one SNP at a time in a linear regression model. The model fit between each SNP genotype and phorbol ester content was then estimated using the program Statistica v.13.0 (Statsoft, Dell).

## Supplementary information

S1,S2,S3,S4
